# Prevalence and Risk Factors of Superficial Fungal Infection among Patients Attending a Tertiary Care Hospital in Central Nepal

**DOI:** 10.1155/2022/3088681

**Published:** 2022-10-04

**Authors:** Vidya Laxmi Jaishi, Ranjana Parajuli, Pragyan Dahal, Roshani Maharjan

**Affiliations:** ^1^Tri-Chandra Multiple Campus, Kathmandu, Nepal; ^2^Grande International Hospital, Kathmandu, Nepal

## Abstract

Fungal infections of hair, nail, and skin are common worldwide and tend to increase. The present study was conducted to determine the prevalence of dermatomycoses, estimate the efficiency of rapid potassium hydroxide (KOH) wet-mount, and observe the hygienic status and the predisposing risk factors. Altogether 115 samples (nail = 77, skin = 30, and hair = 8) were obtained in a duration of December 2019 to June 2020 at Grande International Hospital, Nepal. The samples were examined by KOH wet-mount microscopy and further processed for culture. The dermatophyte test medium (DTM) was used to isolate dermatophytes separately. The fungal colonies obtained in SDA, SDA with cycloheximide/chloramphenicol and dermatophyte medium were subjected to lactophenol cotton blue (LPCB) reagent to study fungal morphology. The yeast colonies grown on SDA were subjected to Gram staining, germ-tube tests, and biochemical tests for identification. CHROMagar was used to distinguish different *Candida* species based on its pigment production in the medium. Various factors (age, sex, occupation, and hygiene condition) were analyzed which were associated with mycological infection. Out of 115 samples, the presence of fungal elements was detected in 20 samples by KOH. Nondermatophyte molds were the most isolated fungus in nails, skin, and hair, followed by yeast and dermatophytes, respectively. Dermatomycosis molds were the most common causative agents with 22 (14.7%) cases, followed by yeasts with 6 (5.21%) cases. *Candida albicans* was isolated from 5 (4.3%) cases, whereas *Rhodotorula species* accounted for a single (0.8%) case. Dermatophytes were isolated from 5 (4.3%) cases. Among them, *n* = 4(3.4%) cases revealed *Trichophyton rubrum* and *Trichophyton mentagrophytes* was isolated from single (0.8%) case. The most isolated nondermatophyte mold that follows criteria as a pathogen in our study was *Cladosporium species* 6 (25%) out of 27 total fungal isolates. Poor hygiene and sweating were found to be statistically significant (*P* < 0.05) in fungal cases detected by both KOH and culture. Dermatophytes and nondermatophyte fungi were emerging as important causes of fungal infection. Both direct microscopy and culture followed by LPCB together were vital tools for the diagnosis of fungal infections.

## 1. Introduction

Fungal disease is classified into three broad groups: superficial mycosis, subcutaneous mycosis, and systemic mycosis. Superficial infections are the most common fungal infections and are prevalent mostly in countries with tropical, subtropical, and temperate climate [1–3]. Superficial infections are the fungal infections associated with the outermost layer and its appendages such as nails, skin, and hair.

Dermatophytic infection is known to infect skin, nails, and hair that is also referred to as ringworms or tinea. The skin infections caused by nondermatophytic fungi are known as dermatomycoses [2]. Onychomycosis refers to fungal infection of the nails that leads to thickening, discolouration, and separation from the nail bed, and piedra is one of the many different forms involving hair shafts, in which the small nodules are present that are stuck on to the hair shaft [1]. On the globe, dermatophyte and nondermatophyte fungi are recorded everywhere but with variation in distribution, incidence, epidemiology, clinical manifestations, and target hosts from one location to another [4].

In Nepal, the epidemiology of superficial fungal infections is less studied compared to other infections. Lack of health facilities, poor socioeconomic status, and poor hygiene practices like exchanging footwear in developing countries have been recognized as potential risk factors for the existence of the disease [5]. The existence of comorbidities such as diabetes that led to the suppression of the host immune system by underlying diseases made patients more susceptible not only to pathogenic fungi but also to all fungi that were once recognized as contaminants [6, 7]. Hence, this study was undertaken to search out the clinical mycological approach, the prevalence of various clinical varieties of dermatomycoses including dermatophytes, yeast, and mold infections of skin, nails, and their etiological agents.

## 2. Materials and Methods

### 2.1. Ethical Approval and Patient Consent

The ethical clearance was obtained from the Nepal Health Research Council (NHRC). Approval number 198/2020 MT, Kathmandu, Nepal. Patient consent was taken both verbally and in written form.

### 2.2. Study Area and Design

The study was carried out in a tertiary care hospital, Grande International Hospital, Kathmandu, Nepal (27.75°N, 85.33°E) located in Dhapasi at Province No 3. Grande International Hospital is a 200-bed tertiary health care center in Nepal. The hospital building is 15 stories high, sprawling over an area of 29,899 sq. ft. The maximum and minimum temperatures 35°C and 2.4°C, respectively. A prospective cross-sectional study was conducted in a tertiary care hospital in Nepal within the Department of Microbiology between December 2019 to June 2020. The study concerned patients attending the “out-patient department” (OPD) of Grande International Hospital. A total of 115 patients were enrolled in the study that was referred by the dermatologist for the mycological diagnosis following the convenient sampling technique. The samples were collected by medical laboratory technologists with the guidance of a microbiologist in a sample collection zone of the microbiology laboratory.

The inclusion criterion was as follows: Patients of all ages visiting the Dermatology department for suspicion of superficial fungal infection in which the specimens such as nail clipping (*n* = 77), hair (*n* = 30), and skin (*n* = 8) referred to microbiology laboratory were included.

The exclusion criterion was as follows: The patient using antifungal therapy (oral as well as topical creams) within 2–3 days before giving the samples and patients receiving treatment for subcutaneous and deep fungal infections were excluded from the study.

### 2.3. Sample Size Determination

The sample size was calculated using the following formula:

(1) $$\begin{array}{c} N=Z^{2}PQ/d^{2}, \end{array}$$

where *Z*-score = 1.96 at 95% confidence level, *P* = expected prevalence, and *d* = precision or margin of error. Applying a 95% confidence level value of *Z* = 1.96, the expected prevalence of dermatophytes was found to be 6.7% analyzing the past studies [8], and precision (d) = 5%. The sample size was calculated as 97. Adding 18% in the case of unlabelled, mishandled, and inadequate quality specimens = 18% of 97 = 18. Therefore, the estimated sample size in our study was 115.

### 2.4. Sample Collection Procedure for Various Superficial Infections

#### 2.4.1. Nail and Skin Infection

The affected areas of the nail were cleaned with 70% v/v ethanol. The outermost layer was removed by scraping with a scalpel blade; any discolored or brittle part of the nail was clipped and collected in a sterile container. In addition, deeper scraping and debris under the edge of the nail were also collected for better sensitivity. The nails were collected on a clean piece of plain folded paper to increase the visibility of samples [9, 10]. For skin samples, the affected areas of the skin were disinfected with 70% v/v ethanol. Skin epidermal scales were collected by scraping the surface of the margin at the active border of the lesion using a sterile blunt scalpel, and the specimens were kept in a dry sterile container. The samples were transported immediately to the laboratory at room temperature [10].

#### 2.4.2. Hair Infection

Samples were collected by scraping the scalp with a blunt scalpel (that may include hair stubs, skin scales, and contents of plugged follicles). Infected hair was also plucked from the scalp with forceps and kept in a clean, dry sterile container, and the specimens were transported immediately to the microbiology laboratory [10].

### 2.5. Processing and Observation of Different Specimens for KOH Wet-Mount, Culture, and Lactophenol Cotton Blue Staining

#### 2.5.1. KOH Mount

The specimens such as nails, skin scrappings, and hairs were subjected to 20% KOH. Preheating of the slide was done before a microscopic examination when necessary, and the specimens were observed microscopically by applying a thin glass cover slip. The KOH-treated specimens on a glass slide were examined using 10 X and 40 X objectives in a light microscope. The contrast was increased during microscopic examination by omitting a microscope condenser. The microscopic examination was done for observation of hyaline, branched, and septate/aseptate fungal hyphae including yeast cells [11, 12].

#### 2.5.2. Identification of Dermatophytes and Nondermatophytes Molds

The media used for culture were Sabouraud Dextrose Agar (SDA) with and without cycloheximide/chloramphenicol, dermatophytes test medium (DTM), and CHROMagar for yeast cells. The first one was used for all the samples while the second one was used for isolation of dermatophytes species. Second, yeast isolates are applied on CHROMagar while suspected dermatophytes are inoculated on DTM for further confirmation. The inoculated medium was incubated at room temperature (25–30)°C for 3–4 weeks. The identification was done based on colony morphology, growth rate, and pigmentation of dermatophytes and nondermatophytes on the obverse and reverse side of the SDA medium and fungal stain microscopy was done in 40 X magnification using lactophenol cotton blue to observe fungal morphology (such as macroconidia, microconidia, fruiting bodies, and fungal hyphae) [13].

#### 2.5.3. Conditions or Criteria for Implicating Isolated Fungus as Pathogens in a Fungal Culture from Superficial Sites

Isolation of fungal colony in a culture was not easy to interpret especially in nondermatophytes, as there are contaminants of dermatology samples. In general, the clinical significance of nondermatophytes relies on their visualization by microscopic examination. The criteria for considering the isolated fungus in a culture as pathogens in superficial infection were described by bormans and Johnson [14] are as follows:

1. Dermatophytes are significant if isolated in a culture.
2. The clinical significance of isolation of nondermatophyte mold in a culture depends upon detection of fungal elements in clinical samples by direct microscopy that demonstrates intact hyphae.
3. The nondermatophyte mold must be isolated in pure culture from several pieces of specimens in the absence of dermatophytes.
4. The clinical significance of *Candida species* in nail specimen is significant if isolated from paronychia or if seen in direct microscopical examination of specimen for skin specimen. However it depends upon clinical presentation. Other yeasts are unlikely to be significant unless a large amount of budding yeast cells are observed in direct microscopy of a clinical specimen.

#### 2.5.4. Identification of Yeast Cells

Specimens were inoculated on SDA and incubated at 37 C for 24–48 hours. The colony morphology (creamy, pasty colonies) and pigmentation were observed. Gram staining was performed by preparing smears from a direct colony on SDA to observe budding yeast cells as well as their pseudohyphae that retain purple color. Further, a germ tube test was done to distinguish *Candida albicans/dubliniensis* from other non-albicans species. The germ tube test was carried out by inoculating yeast colonies in 0.5 ml serum followed by 2 hours incubation. The presence of germ tubes without constriction in hyphae determines the majority of yeast like *Candida albicans/dubliniensis*. For the identification of *Rhodotorula species,* the colony morphology and pigmentation (pasty and orange colony) were observed on SDA and were further subjected to Gram staining and biochemical tests suggested by Begum et al. [15, 16]. In addition, CHROMagar (also known as a chromogenic medium for yeast) was used to further verify *Candida albicans/dubliniensis* characteristics in germ tube tests and to identify different *Candida species* other than albicans based on its pigment production (figure) by enzyme-substrate reaction in the medium [17] as shown as follows: *C. albicans*-green. *C*.*tropicalis*-blue. *C. krusei*-pink, fussy, and other species-white to mauve.

### 2.6. Statistical Analysis and Software Used

The data were analyzed by applying simple descriptive statistics on demographic data. In addition, the chi-square test was applied to determine sensitivity, specificity, positive predictive value, negative predictive value, and accuracy of the KOH test for fungal diagnosis. The inter-rate reliability was determined using the Kappa agreement. The *P* values and odds ratio were calculated using binomial logistic regression analysis for each dichotomous variable for risk factors using the SPSS version 11.

### 2.7. Results

The total number of patients involved in our study was 115. Altogether *n* = 68 (59.13%) males and 47 (40.86%) females suspected of fungal infection were referred for fungal examination. The mean age group in year ± standard deviation (SD) amongst referred groups was found to be 47 ± 8.5. Among agriculture occupations of various referred participants of the suspicious group the highest number of individuals were involved in poultry farming (8.6%) followed by livestock farming (7.8%) and crop farming (5.2%), as shown in Table 1.

Out of 115 suspected participants, tinea corporis was found to be predominant in our study, i.e., 38 (36.51%) cases between ages of 21–50 years in both sexes followed by tinea unguium 39 (34.78%) between ages of 51–70 years in both the genders. Tinea manuum was the least observed clinical manifestation seen in an elderly patient (81–90 years). The results are summarized in Table 2:

#### 2.7.1. Results of KOH, Culture, and Lactophenol Cotton Blue Identification Assay

**Figure 1:**
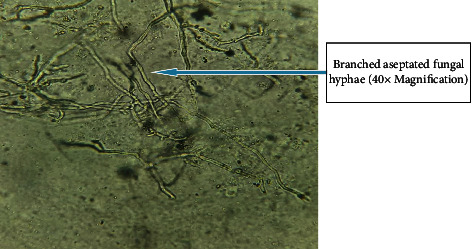
KOH wet-mount of skin sample showing hyaline, branched, aseptate fungal hyphae.

**Figure 2:**
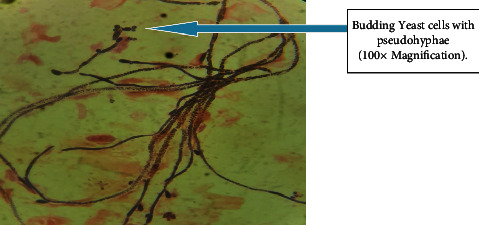
Budding yeast cells with pseudohyphae observed in Gram staining.

**Figure 3:**
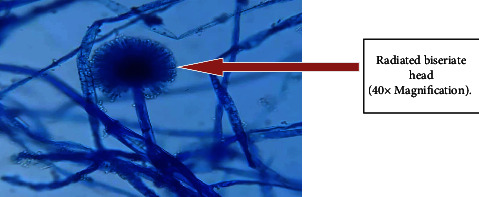
*Aspergillus flavus* showing structural radiated biseriate head in LPCB stain.

**Figure 4:**
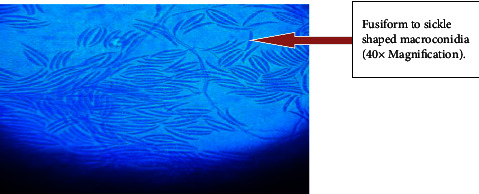
Fusiform to sickle shaped macroconidia of *Fusarium species* in LPCB stain.

**Figure 5:**
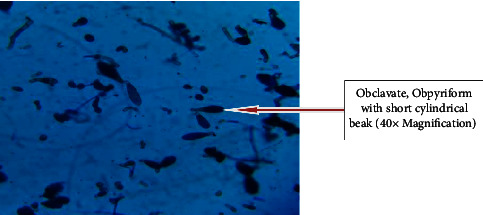
*Alternaria* spp (macroconidia: Obclavate, obpyriform, often with short conical or cylindrical beak, pale brown) in LPCB stain.

Out of 115 specimens subjected to KOH wet-mount for the diagnosis of fungal infection, 26 (22.60%) specimens were positively diagnosed by direct microscopic examination as shown in Figure 1. Among them *n* = 16 (15.6%) was observed as branched septate fungal hyphae, *n* = 1 (0.8%) was observed as branched aseptate hyphae (Figure 1), and *n* = 3 (2.6%) was observed as budding yeast cells by direct microscopic examination under KOH wet-mount. Similarly, 33 (28.69%) fungal cases were diagnosed by culture using SDA, SDA with chloramphenicol, dermatophyte medium, and CHROMagar. Amongst 33 fungal cases, 7 colonies were found to be yeast cells which were confirmed by first observing plenty of budding yeast cells in direct KOH examination of clinical samples as well as observing creamy pasty colonies and pigment production on SDA. The direct colony of yeast was subjected to Gram staining to observe budding yeast cells (Figure 2) and was confirmed further by CHROMagar (green colonies). Altogether out of 33 culture-positive samples, 26 growths of filamentous fungi were observed in culture. Amongst them, KOH wet-mount detected hyphae of 23 filamentous fungi. Further identification was done using LPCB wet-mount staining to observe fungal elements such as radiated biseriate heads (Figure 3) and macroconidia in Figure 4 and Figure 5.

Altogether, *n* = 20 (17.39%) fungal elements were detected by both the methods KOH wet-mount as well as culture. Amongst them, *n* = 16 (13.9%) specimens were positive for nondermatophyte molds in KOH wet-mount and *n* = 3 (2.6%) yeast cells were detected in direct microscopic examination of clinical specimens, whereas *n* = 5 (4.3%) dermatophytes were detected only by culture following the LPCB identification. Therefore, the characteristics of detected nondermatophyte mold in both the culture and the KOH method that follows Borman's criteria [14] were illustrated below in Table 3:

#### 2.7.2. Evaluation of Diagnostic Accuracy of KOH Wet-mount Microscopy vs. Culture

By using fungal culture as a reference method, 33 participants were positive for superficial mycoses while altogether 82 participants were negative for superficial mycoses. KOH wet-mount microscopy correctly identified 20 superficial mycoses cases. The sensitivity and specificity of KOH wet-mount microscopy were 71.7%, 95%CI (56.5% to 84.0%), and 93.1%, 95% CI (85.7% to 97.4%), respectively. The KOH wet-mount microscopy kappa value of 0.5, 95% CI (0.7 to 0.4) has good agreement when compared to the fungal culture. Furthermore, the positive predictive value (PPV) and negative predictive value (NPV) of KOH wet-mount microscopy were 84.6%, 95% CI (71.3% to 92.4%), and 86.3%, 95% CI (79.8% to 90.9%), respectively. Even though the performance of KOH wet-mount microscopy had less sensitivity and specificity compared to fungal culture, the overall accuracy of KOH wet-mount microscopy was found to be 85.8%, 95% CI (78.7% to 91.2%) which was statistically significant (*P* value <0.001) as shown in Table 4.

#### 2.7.3. Fungal Isolates in Nail, Skin, and Hair

**Figure 6:**
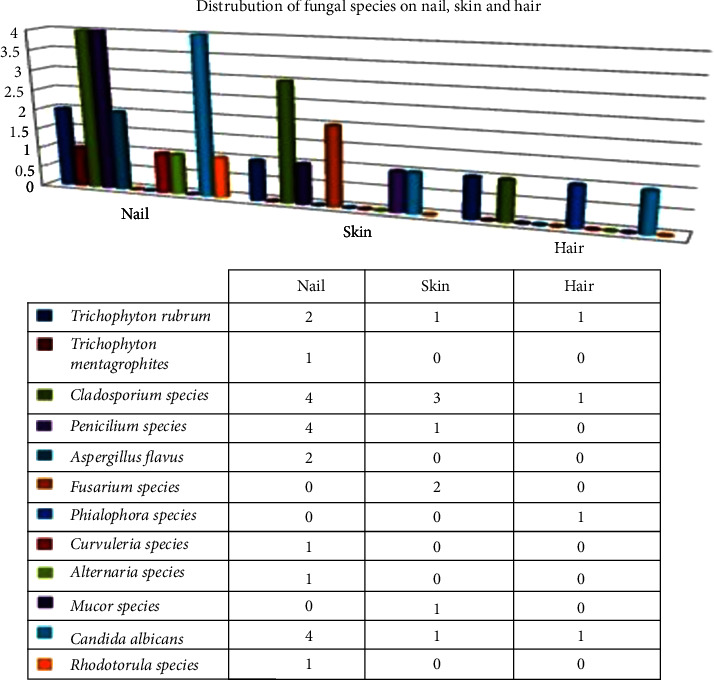
Frequency of fungal isolates from nail, skin, and hair amongst culture-positive cases.

Different species of dermatophytes and nondermatophytic fungal species were isolated from nails, skin, and hair. Nondermatophytes were isolated in the highest proportion *n* = 28 (84.84%). All together *n* = 5 (15.15%) dermatophytes were isolated from nails, skin, and hair. The results depicted in Figure 6 show the distribution of a wide range of dermatophytes and dermatomycosis fungus isolated from nail, skin, and hair.

**Distribution of dermatophyte and nondermatophyte fungi based on significant criteria in culture with the relation to clinical manifestation.**

Among a total of 33 isolates in a fungal culture, 27 fungal isolates were found to be clinically significant following Borman's criteria for culture interpretation. Nondermatophytes mold was the most common causative agent of superficial fungal infection in our study with 16 (13.9%) cases. The most significant nondermatophyte mold causing superficial mycoses was *Cladosporium species* 6 (21.4%) of the total significant isolates in culture, followed by the yeast cells (*Candida albicans*) 5 (18.5%), and one *Rhodotorula species* isolated among the total significant isolates. The majority of yeast cells 5 (18.5%) out of a total of 27 isolates were isolated from tinea unguium. In addition, *Aspergillus flavus*, *Fusarium species,* and *Penicillium species* caused 7.1% of significant mycological infection among total significant culture isolates. Altogether 5 (17.7%) dermatophytes are observed among the total fungal isolates. The highest frequency of dermatophytes isolated in the culture was *Trichophyton rubrum* from patients with tinea unguium followed by tinea curis and tinea corporis, respectively. One *Trichophyton mentagrophytes* was yielded by Tinea corporis. Observing all the significant fungal isolates, the highest number of fungus was isolated from patients with tinea corporis in our study. The results are summarized in Table 5.

**Figure 7:**
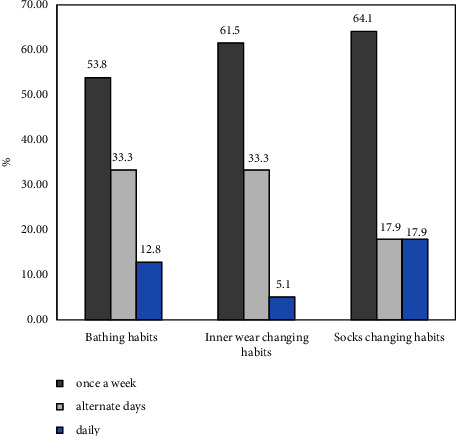
Hygienic status of positive cases.

The hygiene status of the positive cases was noted in our study via questionnaires like bathing, replacement of inner wear, and clothing as well as sock changing habits. Most fungal positive cases were seen in patients who took baths once a week (53.8%), followed by alternate days (33.3%), and regularly (12.8%). Similarly, the majority of yeast infections were observed from skin specimens and culture amongst patients with poor inner-wear changing habits. The higher risk of fungal infection in the foot (tinea pedis) was amongst those participants who had poor socks hygiene. Minor cases were found in patients who had good hygiene practices, as shown in Figure 7.

Statistical analysis was performed on risk factors by applying binary logistic regression analysis to two dichotomous variables. Risk factor analysis results showed that there was a statistically significant association between the existence of positive fungal cases and the sweating nature of skin (OR = 9.48, *P* < 0.00001). Amongst all the three risk factors, sweating nature had 10 times the odds of having superficial fungal infections compared to other risks factors followed by poultry and cattle farm occupation (Table 6). Poultry occupation and knowledge of fungal infection had the least odds that contributed to the risk of positive cases and were found to be statistically insignificant (*P* > 0.05).

## 3. Discussion

The present study shows that the suspicious cases of superficial fungal infection represent various clinical manifestations and was familiar with the mean age group of 47 years. The study shows a similar pattern to another study done by Mercentini et al. [18] in which the most commonly infected age group was above 40 years. Thus, the highest incidence of infection in this age group could be due to the increased physical activity and increased opportunity for exposure. Males (59.1%) were more commonly affected than the females (40.8%). The high incidence of males might be due to more outdoor exposure [19] and the predominant number of occupational exposures in males was further determined by the study of Tartor et al. [20] that has similar trends with our study.

In our study, seven different types of tinea were observed, amongst which tinea corporis was the predominant clinical manifestation that accounts for 36.51% followed by tinea unguium (34.78%) of the total cases. The prevalence obtained in our study can be compared with the other published studies [19, 21]. Out of 115 patients, fungal isolates were observed in 33.91% , indicating that differentiation of fungal infections only based on clinical means is unreliable. Based on fungal diagnosis, KOH wet-mount microscopy identified 20 cases of superficial mycoses. The sensitivity and specificity of KOH wet-mount microscopy, when compared with culture, was found to be 71.74%, 95% CI (56.54% to 84.01%) and 93.18%, 95% CI (85.75% to 97.46%), respectively. A similar range of sensitivities of the KOH test was observed in a study conducted by Levitt et al. [22] where the sensitivities of KOH wet-mount microscopy in their study were 73.3%, 95% CI (66.3–79.5) when observed concerning clinical cure, as well as the specificity was lower compared to the culture with respect to clinical cure. Similarly, diagnosis of onychomycosis by the KOH method in a study conducted by Summer bell et al. [23] reported 73% sensitivity. These two studies yielded the exact sensitivity of the KOH test to our study. Nevertheless, there exist discrepancies between the culture and KOH results like Arabatzis et al. [24] designated 92 samples (skin, nails, and hairs) from 67 participants with suspected dermatophytosis and observed the superiority of the KOH test over fungal culture (43% vs 33%). These findings were consistent with the other studies where KOH diagnosis of fungal infections was found to be culture negative due to dead fragments of fungal hyphae or unviable organisms in the fungal culture medium [19, 25]. In addition, false negativity in KOH occurs due to its sensitivities, inadequate specimen, low dissolvement of keratin of clinical specimens, and unexperienced microscopist [22–25]. Therefore observing various published research [23–26] that concluded culture should be implemented to know viable organisms and active infection of fungus, our study determined culture as a reference method when compared with the KOH wet-mount microscopy.

Amongst the dermatophytes, *Trichophyton rubrum* is noted as the predominant species of dermatophytes in our study. Several other reports have determined *Trichophyton rubrum* as the major dermatophyte species followed by *Trichophyton mentagrophytes* [20, 26]. Nondermatophytic molds account for 84% of the total culture-positive fungal infection in our study. Nails and skin were the most affected region. Our study was consistent with the findings of fungal infection reports done by Greer [27], in which the incidence of nail infections or onychomycosis was found to be 53% of the total cases. The skin infection caused by nondermatophytic molds has also been depicted in a wide range of research studies [23, 25–30]. The highest isolation of nondermatophytes in our study was *Cladosporium species* which accounted for 24.24% of the total cases, and out of them, 5. The study conducted in Ethiopia has revealed the maximum isolation of *Cladosporium species* amongst nondermatophytes [31].

A similar study in Ethiopia done by Arya et al. [9] also yielded the highest isolates of *Cladosporium species* amongst nondermatophytes. Several reports and studies have also concluded that they are recognized as an opportunistic infection in subcutaneous form, especially amongst patients with low immune status [32–35]. *Candida albicans* were isolated mostly in onychomycosis. The existence of these pathogens that lead to tinea infection and onychomycosis has been supported by other studies [34, 36].

In a polluted and big crowded city, the hygiene status of the patients plays a major role in overcoming the fungal infection. The majority of fungal positive cases were seen in patients who took bath once a week (53.84%), and the least cases were observed in those that practiced cleanliness regularly (12.82%). Similarly, skin infections were observed in those patients with poor innerwear and socks changing habits. Likewise, sweating nature has a significant role in promoting fungal infections of both dermatophytes and nondermatophytes which showed a significant association between them (*P* < 0.001). One study done in Nepal showed a similar trend to our study where sweating nature resulted in 3.20 times higher risk with tinea infection as well as the odds of getting fungal infections were high amongst those patients with poor hygiene status [25]. Besides that, occupational contact with the cattle and poultry farm plays a major role in spreading fungal infections. The identified fungal species from cattle and poultry farmers in our study were *Candida albicans, Cladosporium species, Aspergillus flavus,* and *Trichophyton mentagrophytes*. Different domestic animals like cattle, horses, pigs, and cats, as well as birds, are susceptible to *Candida* infections [37, 38]. This determines the possibilities of animals that could be the vectors of transmission or potential reservoirs causing human disease and may represent a risk to the immunocompromised patients. Although, tremendous cases of candidiasis in animals are published in several literature searches [38–40], scarce information is known about its origin and identities of these infecting strains and the genetic relationship that exists among C. albicans isolated from the human and animal sources [41]*. Cladosporium species* account for the highest isolated fungus amongst poultry breeders in our study. Despite its potential pathogenicity in chickens is not known, however, it has been reported that *Cladosporium herbarum* produces dermatitis in chickens. Similarly, this has been further supported by several studies where the isolation of *Cladosporium species* in poultry stands at the highest in many other studies [42, 43]. Furthermore, *Aspergillus species* are also found to be responsible for causing aspergillosis in chickens [43]. Regarding dermatophytes (*Trichophyton mentagrophytes*), they were isolated from the cattle farmers in our study. The cases of dermatophytosis in cattle remain complicated to eradicate due to a wide range of factors such as antifungal resistance, shortage of authorized antifungal agents for the application in veterinary practices, and issues in systemic treatment of cattle because of hepatotoxicity. Spread in both humans and cattle is due to asymptomatic carriers of dermatophytes that were discussed in the study conducted by Tartor et al. [44].

## 4. Conclusion

Both culture and direct microscopy were equally important for the diagnosis of mycoses as culture can determine infection in an active state, whereas KOH wet-mount is rapid and effective in screening for fungal infections from various sites of the body. Hence, both tests are required to be done simultaneously to diagnose fungal infection. The presence of an enormous number of nondermatophyte fungi in our study showed that nondermatophyte fungi are emerging as important causes of dermatomycoses. Hence, the nondermatophytes should not be vastly underestimated. Various physical factors were found to cause the rise of superficial fungal infections like sweating nature, dress changing patterns, and cattle and poultry farmers in our study. Therefore, every individual should strictly follow hygiene and cleanliness practices with a point of care to minimize the risk of fungal infection.

## Figures and Tables

**Table 1:** Demographic characteristics of study population.

Characteristics	Patients
Mean age in years (SD)	47 ± 8.5
Gender	
Males (%)	68 (59.1)
Females (%)	47 (40.6)
Occupations	
Livestock farming (%)	9 (7.8)
Poultry farming (%)	10 (8.6)
Crop farming (%)	6 (5.2)
Others	90 (78.2)
Total (%)	115 (100)

**Table 2:** Age and sex-wise distribution of participants based on clinical manifestation.

Age (in years)	Male	Female	Total (%)	Clinical manifestation
≤10	1	1	2 (1.7)	Tinea incognita
11–20	7	5	12 (9.5)	Tinea faciei
21–30	5	3	8 (6.9)	Tinea corporis
31–40	10	7	17 (14.7)	Tinea cruris
41–50	19	15	34 (29.5)	Tinea corporis
51–60	13	6	19 (16.5)	Tinea unguium
61–70	11	9	20 (18.2)	Tinea unguium
71–80	1	1	2 (1.7)	Tinea capitis
81–90	1	0	1 (0.8)	Tinea manuum
Total	68	47	115 (100)	

**Table 3:** Direct microscopic examinations results of nondermatophytes mold with colony characteristics in SDA with their LPCB identification.

KOH wet-mount results/Direct microscopic examination	Number of fungal elements observed in direct KOH wet-mount.	Fungal colony morphological characteristics in SDA	LPCB identification
Dark, branched septate fungal hyphae seen.	6	Olivaceous-brown to blackish-brown colonies suede-like to floccose turning later into powdery colony due to the production of abundant conidia. Olivaceous-black pigments in the reverse side.	*Cladosporium species*
Hyaline, branched septate fungal hyphae seen.	2	Green, velvety, wrinkled colony. Not any pigmentation observed in the reverse side.	*Penicillium species*
Hyaline, branched septate fungal hyphae with acute angle branching seen.	2	Granular, flat colonies with radial grooves, yellow at beginning later quickly becoming bright to dark yellow-green. Lemon yellow pigment in reverse side	*Aspergillus flavus*
Hyaline, branched septate fungal hyphae with acute angle branching seen.	2	Pale, bright-colored, fast-growing colonies with a cottony aerial mycelium. No pigmentation was observed in reverse side.	*Fusarium species*
Brown branched septate fungal hyphae seen.	1	Woolly to velvety colony, suede like and olivaceous to black in obverse and reverse side.	*Phialophora species*
Dark brown pigmented, branched septate fungal hyphae seen.	1	Dark brown and velvety, loose cottony at the center with black pigmentation in reverse side.	*Curvularia species*
Dark, branched septate fungal hyphae seen with branched chains of large conidia.	1	Colonies appeared black to olivaceous-black or grayish and has suede-like to floccose appearance.	*Alternaria species*
Hyaline, branched aseptate fungal hyphae seen.	1	White to grayish brown, fast-growing colonies resembling salt and pepper appearance.	*Mucor species*
Plenty of budding yeast cells seen. (Arthroconidia was observed in LPCB mount and Gram staining prepared from culture)	3	Soft, smooth, mucoid, and orange pigmented colony in the obverse side.	*Rhodotorula species*
		White, small, creamy, and pasty colony	*Candida species*

**Table 4:** Evaluation of KOH preparation with culture for the detection of fungal infection.

Test	Culture			
KOH		Positive	Negative	Total
	Positive	20 (TP)	6 (FP)	26
	Negative	13 (FN)	76 (TN)	89
		Total = **33**	Total = **82**	Total = **115**
Sensitivity	71.7%, 95% CI (56.5% to 84.0%)			
Specificity	93.1%, 95% CI (85.7% to 97.4%)			
PPV	84.6%, 95% CI (71.3% to 92.4%)			
NPV	86.3%, 95% CI (79.8% to 90.9%)			
Accuracy	85.8%, 95% CI (78.7% to 91.2%)			
Kappa value	0.5, 95% CI (0.7 to 0.4)			
*P* Value	<0.001			

Abbreviations. TP = true positive; FP = false positive; TN = true negative; FN = false negative.

**Table 5:** Distribution of fungal isolates concerning clinical manifestation.

Fungal isolates	Clinical presentation							
	Tinea capitis	Tinea unguium	Tinea faciei	Tinea cruris	Tinea manuum	Tinea corporis	Tinea cruris	Total (%)
*Trichophyton rubrum*		2		1		1		4 (14.2)
*Trichophyton mentagrophytes*						1		1 (3.5)
*Cladosporium species*	1	1	1	1		2		6 (21.4)
*Penicillium species*				1		1		2 (7.1)
*Aspergillus flavus*		2						2 (7.1)
*Fusarium species*					1	1		2 (7.1)
*Phialophora species*						1		1 (3.5)
*Curvularia species*		1						1 (3.5)
*Alternaria species*						1		1 (3.5)
*Mucor species*						1		1 (3.5)
*Candida albicans*		4					1	5 (18.5)
*Rhodotorula species*		1						1 (3.5)
*Total*	1 (3.7%)	11 (40.7%)	1 (3.7%)	3 (11.1%)	1 (3.7%)	9 (33.3%)	1 (3.7%)	27 (100)

**Table 6:** Binary logistic regression analysis of risk factors associated with increased fungal infection.

Risk factors associated with increased fungal infection.	Presence of risk factor among fungal positive cases (%)	Absence of risk factor among fungal positive cases (%)	Adjusted odds ratio	*P* value
Sweating nature of skin	23 (85.18)	4 (14.81)	10	<0.001
Poultry and cattle farm occupation	8 (29.62)	11 (40.74)	3	0.08
Knowledge on fungal infection	9 (33.33)	18 (66.66)	1	0.7

## Data Availability

Most of the data generated in our experiments were included in the results section. However, additional raw data can be obtained upon request from the corresponding author (pragyandahal55@gmail.com).

## Additional Points

*Limitations of the Study*. Due to lower number of cases referred by Dermatology department, there is limited sample size in our study. The lack of available resource in a resource limited laboratory settings and not any source of funding. We were unable to conduct molecular assays simultaneously with the culture and KOH methods.
